# Analysis of factors associated with the development of delayed encephalopathy following acute carbon monoxide poisoning

**DOI:** 10.1038/s41598-024-64424-7

**Published:** 2024-06-25

**Authors:** Zi-bo Liu, Li-chun Wang, Jia-jia Lian, Sha Li, Long Zhao, Hong-Ling Li

**Affiliations:** 1 Department of Endocrinology, The Second Hospital of Hebei Medical University, Shijiazhuang, 050000 People’s Republic of China; 2 Rehabilitation Department, Cangzhou Hospital of Integrated Traditional Chinese and Western Medicine, Cangzhou, 061001 Hebei People’s Republic of China; 3 Department of Rehabilitation, The Second Hospital of Hebei Medical University, No215 of Hepingxi Road, Xinhua District, Shijiazhuang, 050000 Hebei People’s Republic of China

**Keywords:** Acute carbon monoxide poisoning, Clinical characteristics, Efficacy of hyperbaric oxygen therapy, Influencing factors, Prognosis, Biochemistry, Biotechnology

## Abstract

In this study, we analyzed the factors influencing the development of delayed encephalopathy in patients with acute carbon monoxide poisoning (ACOP) (DEACMP) following conventional treatment such as hyperbaric oxygen therapy (HBOT). Between January 2012 and January 2022, we retrospectively analyzed 775 patients with ACOP, who were admitted to the Second Department of Rehabilitation Medicine and received HBOT in the Second Hospital of Hebei Medical University. These patients were divided into the non-DEACMP and DEACMP groups based on their follow-up; we then compared the general data, clinical characteristics, admission examination, and treatment between the two groups to identify risk factors for the development of DEACMP. The DEACMP group comprised of 168 cases, while the non-DEACMP group consisted of 607 cases. Univariate analysis showed that there were 20 possible prognostic factors in the non-DEACMP and DEACMP groups. The results of multivariable regression analyses suggested that the occurrence of DEACMP was significantly correlated with advanced age, the combination of multiple medical histories, the duration of CO exposure, the duration of coma, poisoning degree, the Interval between ACOP and the first HBOT, the total number of HBOTs, and the combination with rehabilitation treatment. DEACMP patients who are older, have more comorbidities, prolonged CO exposure, prolonged coma, severe intoxication, long intervals between ACOP and the first HBOT, fewer HBOT treatments, and who are not treated with a combination of rehabilitative therapies have a poor prognosis.

## Introduction

Carbon monoxide (CO) is an odorless and colorless, non-irritating toxic gas that is the most prevalent asphyxiant gas in industrial production and living environments^1^. Consequently, acute carbon monoxide poisoning (ACOP) continues to be one of the most common and lethal acute diseases worldwide. ACOP has an estimated global incidence rate of 137 cases per million people and a global mortality rate of 4.6 deaths per million people, with epidemiological characteristics varying by country^2,3^. Several factors, including the occurrence of delayed encephalopathy in patients with ACOP (DEACMP), can influence the severity of symptoms and disease outcomes of ACOP. ACOP has a poor prognosis and places a significant burden on society and families. Therefore, early identification and prediction of DEACMP is of great significance. A multicenter study showed that age, duration of CO exposure, and GCS score were independent risk factors for DEACMP in ACOP patients^4^. Domestic and international studies have shown that age, gender, occupation, severity of acute-phase poisoning, duration of coma (i.e., the time from when the patient falls into a coma until the patient wakes up), cranial imaging abnormalities, hyperbaric oxygen treatment measures, complications, and post-poisoning mental stimulation are associated with the occurrence of DEACMP^5–8^, but due to the small sample size or the influence of factors such as the study population and research methods, different opinions and conclusions have been generated. Large samples and high-quality multi-center randomized controlled studies are still needed in the future. DEACMP has a long course and poor prognosis, HBO is often used to treat DEACMP clinically^9^. A systematic evaluation in 2019 showed that HBO treatment can effectively improve the clinical symptoms of DEACMP patients^10^. There are fewer foreign reports on HBO treatment for DEACMP, and there is a lack of large-sample clinical studies. Although HBO treatment for DEACMP has achieved good efficacy, there is no unified conclusion on the therapeutic pressure, duration, and intervention time of HBOT. This study intends to retrospectively analyze the patients with DEACMP treated with HBO in the past 10 years, observe the clinical characteristics of DEACMP patients, and analyze the relevant influencing factors of its occurrence, in order to provide reference for the early prevention of DEACMP and reduce the risk of occurrence.

## Materials and methods

### General data

In this study, we enrolled 1436 patients diagnosed with ACOP and treated with HBOT at the Second Hospital of Hebei Medical University from January 2012 to January 2022. Hebei province is a high-risk area for ACOP, especially in winter, where many people use charcoal burning for heating. After excluding 325 patients failure to meet inclusion criteria or had incomplete data, 206 patients were lost to follow-up, and 130 patients died due to other conditions during follow-up, 775 patients were finally included in the analysis of influencing factors. The inclusion criteria are described as follows: (1) patients with ACOP who visited the Second Department of Rehabilitation Medicine of our hospital and received HBOT treatment between January 2012 and December 2022; (2) people who matched the diagnostic criteria for ACOP and were diagnosed with COP based on a comprehensive medical history and physical examination;^11^ (3) patients who approached our hospital within three days after the onset of the disease; (4) Patients and their families who signed the informed consent form for participating in the study. The exclusion criteria are listed below: (1) patients missing a significant amount of clinical data; (2) individuals who died from different conditions during follow-up; (3) patients who refused to cooperate in the follow-up.

### Methods

All patients underwent conventional treatment (blood pressure, ECG monitoring, oxygen saturation monitoring and symptomatic treatment with medication according to condition) and HBOT (pressure: 2.0 ~ 2.5ATA) while completing rehabilitation therapy (transcranial direct current stimulation, median nerve electrical stimulation, cerebellar parietal nucleus electrical stimulation, low-frequency pulsed electrical stimulation, lower limb intelligent rehabilitation training system, swallowing electrical stimulation) according to individual dysfunction.Data about the participants including their gender (male/female), age, level of education (illiterate/primary school education/secondary school education/above secondary school education), occupation (physical labor: workers, farmers, self-employed persons/brainwork: police, teachers, civil servants, students), medical history (no/hypertension/diabetes/coronary heart disease/cerebral infarction/combination with multiple disease), poisoning method (life poisoning/occupational poisoning), poisoning degree (mild/moderate/severe) (see Appendix), duration of CO exposure, duration of coma, relevant functional scores (Glasgow coma scale, Mini-mental state examination, Modified Barthel Index) at admission, laboratory index values (blood gas analysis, creatine kinase, creatine kinase isoenzyme, electrolyte level), electrocardiogram, results of the first head CT and MRI upon admission (Abnormalities: demyelination or imaging density changes in periventricular white matter, semiovals, corpus callosum knees and presses, internal capsule, pallidum, caudate nucleus, chiasmatic nucleus, dorsal thalamus, substantia nigra, and nucleus accumbens), and Interval between ACOP and the first HBOT, HBOT treatment regimen, and number of treatments, the presence of combined rehabilitation treatment, and discharge status were collected. Patients were followed up after discharge by phone and WeChat to record their post-charge discovery. The presence of DEACMP was detected using the DEACMP diagnostic criteria^11^: After the recovery from consciousness impairment following acute carbon monoxide poisoning, one may experience one of the following clinical manifestations within approximately 2–60 days of a “pseudo-recovery” period:

- Mental and consciousness impairment manifesting as dementia, delirium, or cortical absence state;
- Extrapyramidal nervous system disorders presenting as symptoms of Parkinson’s syndrome;
- Pyramidal system nerve damage (such as hemiplegia, positive pathological reflexes, or urinary incontinence);
- Focal cortical dysfunction such as aphasia, blindness, or secondary epilepsy. Head CT scans may reveal areas of pathological density reduction in the brain; EEG examinations may show moderate to severe abnormalities.

### Statistical analysis

The collected data were entered into an Excel database for initial screening, and the data that satisfied the inclusion and exclusion criteria were processed and analyzed with the SPSS 26.0 software. Measurement data were evaluated using the normality test, the data were examined using the t-test for normal distribution and homogeneity of variance, with the *t*′ test in the case of heterogeneity of variance (mean ± standard error [$\overline{X}$ ± SE] were calculated), and with the non-parametric test when not normally distributed (the quartiles [M ± Q] were calculated). The number of cases and composition ratios in the count data were evaluated using chi-squared test. Relevant influencing variables were subjected to univariate analysis, and variables with statistically significant differences between groups were selected for multivariable logistic regression analyses. Differences were deemed statistically significant at *P* < 0.05.

### Ethics approval and consent to participate

This study was granted exemption by the ethics committee of The Second Hospital of Hebei Medical University. We certify that the study was performed in accordance with the 1964 declaration of HELSINKI and later amendments.

## Results

### Univariate analysis of influencing factors for the occurrence of DEACMP

There were 607 cases in the non-DEACMP group of patients with ACOP and 168 in the DEACMP group. The findings of the univariate analysis of clinical data revealed that the two groups were statistically significantly different in terms of Mini-Mental Status Exam score (Z = − 14.843, *P* < 0.001), modified Barthel index (Z = − 12.224, *P* < 0.001), age (X2 = 93.821, *P* < 0.001), education level (X2 = 42.072, *P* < 0.001), labor type (X2 = 36.538, *P* < 0.001), medical history (X2 = 151.606, *P* < 0.001), poisoning method (X2 = 9.539, *P* < 0.001), duration of CO exposure (X2 = 121.308, *P* < 0.001), duration of coma (X2 = 256.392, *P* < 0.001), presence of coma or not (X2 = 119.414, *P* < 0.001), Glasgow Coma Scale score (X2 = 174.407, *P* < 0.001), poisoning degree (X2 = 262.130, *P* < 0.001), lactic acid level (X2 = 38.971, *P* < 0.001), creatine kinase (CK; X2 = 25.000, *P* < 0.001), CK-MB (X2 = 20.836, *P* < 0.001), electrolyte level (X2 = 21.135, *P* < 0.001), head CT/MRI (X2 = 67.087, *P* < 0.001), Interval between ACOP and the first HBOT (X2 = 84.096, *P* < 0.001), total number of HBOTs (X2 = 244.098, *P* < 0.001), and combination with rehabilitation treatment (X2 = 8.915, *P* = 0.003). There were no marked differences in terms of gender, carboxyhemoglobin concentration, or HBOT pressure between the non-DEACMP and DEACMP groups (*P* > 0.05) (Table 1).

**Table 1:** Univariate analysis of factors influencing the occurrence of delayed encephalopathy.

Influencing factors		Non-DEACMP group (N = 607)	DEACMP group (N = 168)	*P*
MMSE score		30(30,30)	0(0,27)	< 0.001
BMI		95(89,95)	0(0,89)	< 0.001
COHb concentration		16.9(11.1,24.1)	18.8(10.98,29.48)	0.083
Gender	Male	297(77.1%)	88(22.9%)	0.428
	Female	310(79.5%)	80(20.5%)	
Age (years)	< 40 years	285(96.6%)	10(3.4%)	< 0.001
	≥ 40 years	322(67.1%)	158(32.9%)	
Education level	Illiterate	50(60.2%)	33(39.8%)	< 0.001
	Primary school education	424(76.7%)	129(23.3%)	
	Secondary school education	49(92.5%)	4(7.5%)	
	Above secondary school education	84(97.7%)	2(2.3%)	
Labor type	Physical labor	481(74.3%)	166(25.7%)	< 0.001
	Brainwork	126(98.4%)	2(1.6%)	
Medical history	No	525(87.8%)	73(12.2%)	< 0.001
	Hypertension	41(49.4%)	42(50.6%)	
	Diabetes	9(69.2%)	4(30.8%)	
	Coronary heart disease	5(41.7%)	7(58.3%)	
	Cerebral infarction	7(70.0%)	3(30.0%)	
	Combination with multiple diseases	20(33.9%)	39(66.1%)	
Poisoning method	Life poisoning	560(77.1%)	166(22.9%)	0.002
	Occupational poisoning	47(95.9%)	2(4.1%)	
Duration of CO exposure	< 6 h	320(91.7%)	29(8.3%)	< 0.001
	≥ 6 h and < 12 h	266(73.5%)	96(26.5%)	
	≥ 12 h and < 24 h	14(29.2%)	34(70.8%)	
	≥ 24 h	7(43.8%)	9(56.3%)	
Duration of coma	< 6 h	553(90.2%)	60(9.8%)	< 0.001
	≥ 6 h and < 12 h	21(48.8%)	22(51.2%)	
	≥ 12 h and < 24 h	2(10.5%)	17(89.5%)	
	≥ 24 h	31(31.0%)	69(69.0%)	
Presence of coma	No	365(94.6%)	21(5.4%)	< 0.001
	Yes	242(62.2%)	147(37.8%)	
GCS score	3–8 points	45(36.6%)	78(63.4%)	< 0.001
	9–12 points	7(38.9%)	11(61.1%)	
	13–15 points	555(87.5%)	79(12.5%)	
Poisoning degree	Mild	345(98.9%)	4(1.1%)	< 0.001
	Moderate	172(82.3%)	37(17.7%)	
	Severe	90(41.5%)	127(58.5%)	
Lactic acid level	Normal	541(82.2%)	117(17.8%)	< 0.001
	Abnormal	66(56.4%)	51(43.6%)	
CK	Normal	574(80.5%)	139(19.5%)	< 0.001
	Abnormal	33(53.2%)	29(46.8%)	
CK-MB	Normal	574(80.3%)	141(19.7%)	< 0.001
	Abnormal	33(55.0%)	27(45.0%)	
Electrolyte level	Normal	552(80.8%)	131(19.2%)	< 0.001
	Abnormal	55(59.8%)	37(40.2%)	
Head CT/MRI	Normal	549(83.4%)	109(16.6%)	< 0.001
	Abnormal	58(49.6%)	59(50.4%)	
Interval between the first HBOT and ACOP	< 6 h	218(92.0%)	19(8.0%)	< 0.001
	≥ 6 h and < 12 h	230(84.2%)	43(15.8%)	
	≥ 12 h	159(60.0%)	106(40.0%)	
HBOT pressure	2.0ATA	557(78.5%)	153(21.5%)	0.430
	2.2ATA	34(81.0%)	8(19.0%)	
	2.5ATA	10(62.5%)	6(37.5%)	
	Others	6(85.7%)	1(14.3%)	
Total number of HBOTs	1–10 times	451(96.0%)	19(4.0%)	< 0.001
	11–20 times	93(60.8%)	60(39.2%)	
	21–30 times	45(49.5%)	46(50.5%)	
	> 30 times	18(29.5%)	43(70.5%)	
Combined rehabilitation treatment	Yes	550(77.0%)	164(23.0%)	0.003
	No	57(93.4%)	4(6.6%)	

### Multivariable analysis of influencing factors for the occurrence of DEACMP

After the confounding factors were excluded, factors with statistically significant differences in the univariate analysis were included in multivariable logistic regression analyses, which revealed age, medical history, duration of CO exposure, duration of coma, degree of poisoning, Interval between ACOP and the first HBOT, total number of HBOTs, and combined rehabilitation treatment as independent influencing factors for the occurrence of DEACMP. Specifically, the risk of DEACMP was 8.436 times higher in patients aged ≥ 40 years compared to those aged < 40 years, 11.47 times higher in patients with a history of multiple diseases compared to those without a medical history, and 6.328 times higher in patients with 12 h–24 h of CO exposure compared to those with < 6 h of CO exposure. The risk of DEACMP was increased by 6.434-fold in patients with ≥ 24 h of coma compared to those with < 6 h of coma, by 16.985 times in severe ACOP compared to patients with mild ACOP, and 7.068 times in patients with ≥ 12 h of Interval between ACOP and the first HBOT compared to those with < 6 h of interval, and reduced by 0.130-fold in patients with combined rehabilitation treatment (Table 2).

**Table 2:** Multivariate logistic regression analysis of factors influencing the occurrence of delayed encephalopathy.

Risk factors	*P*	*OR*	95%*CI* of *OR*
Age	0.008	8.436	1.740, 40.908
Education level			
Illiterate	0.227		
Primary school education	0.056	0.371	0.134, 1.027
Secondary school education	0.947	0.925	0.093, 9.216
Above secondary school education	0.456	0.360	0.025, 5.276
Labor type	0.584	0.533	0.056, 5.056
Medical history			
No	< 0.001		
Hypertension	0.070	2.242	0.935, 5.374
Diabetes	0.127	6.116	0.597, 62.666
Coronary heart disease	0.091	5.987	0.753, 47.572
Cerebral infarction Combination with multiple diseases	0.349 < 0.001	2.504 11.470	0.367, 17.058 4.017, 32.752
Poisoning method	0.343	0.412	0.066, 2.580
Duration of CO exposure			
< 6 h	0.037		
≥ 6 h and < 12 h ≥ 12 h and < 24 h ≥ 24 h	0.280 0.004 0.516	1.597 6.328 1.912	0.683, 3.738 1.793, 22.337 0.270, 13.553
Duration of coma			
< 6 h	< 0.001		
≥ 6 h and < 12 h	0.026	4.202	1.191, 14.825
≥ 12 h and < 24 h ≥ 24 h	< 0.001 0.007	232.308 6.434	16.777, 3216.755 1.677, 24.691
Presence of coma	0.128	0.376	0.107, 1.326
Poisoning degree			
Mild	0.001		
Moderate	< 0.001	22.096	4.303, 113.457
Severe	0.001	16.985	3.434, 84.010
GCS score			
3–8 points	0.200		
9–12 points	0.452	2.125	0.298, 15.130
13–15 points	0.079	11.513	0.753, 175.986
MMSE score	0.298	0.951	0.866, 1.045
BMI	0.645	0.991	0.955, 1.029
Lactic acid level	0.946	1.033	0.403, 2.648
CK	0.315	0.243	0.016, 3.821
CK-MB	0.309	4.364	0.255, 74.786
Electrolyte level	0.912	0.942	0.326, 2.721
Head CT/MRI	0.779	0.887	0.385, 2.045
Interval between the first HBOT and ACOP			
< 6 h	< 0.001		
≥ 6 h and < 12 h	0.011	3.637	1.352, 9.785
≥ 12 h	< 0.001	7.068	2.734, 18.275
Total number of HBOTs			
1–10 times	< 0.001		
11–20 times	< 0.001	20.165	7.634, 53.266
21–30 times	< 0.001	45.660	14.488, 143.903
> 30 times	< 0.001	40.653	11.832, 139.684
Combined rehabilitation treatment	0.004	0.130	0.032, 0.525

## Discussion

ACOP is a form of poisoning caused by the inhalation of CO produced by the incomplete burning of a large quantity of carbon-containing materials^12^. Between 10–30% of patients with ACOP develop DEACMP, a neuropsychological impairment that occurs after a brief improvement in ACOP symptoms or an asymptomatic interval. These patients demonstrate acute symptoms such as memory loss, motor dysfunction, psycho-behavioral abnormalities, dysgnosia, and bladder/bowel dysfunction 2 days to 1 year after regaining consciousness. The pathogenic mechanisms of DEACMP have been extensively explored, such as ischemic-hypoxic damage, inflammatory and immunological cytokine stimulation, apoptosis, and neurotoxicity due to CO toxicity. However, its pathogenesis remains undetermined. Therefore, there is no definitive treatment modality against etiology, and the present clinical treatment is primarily symptomatic^13–15^.

In this study, the incidence of DEACMP was estimated to be 21.68% during follow-up, and most patients presented symptoms after a pseudo-healing period of 2–52 days following acute poisoning, which is essentially consistent with the results of a prior study^13^. In our study, symptoms improved in 47 patients after 1 to 3 sessions of HBOT, while 121 patients continued to have a poor prognosis or even died. Among the patients with poor prognosis, 14 patients did not continue HBOT after being discharged, and the majority of these patients died from coma and problems connected to prolonged bed rest. Therefore one of our research priorities was to explore factors linked with the occurrence of DEACMP for targeted prevention, to enhance the long-term prognosis of patients with ACOP. Based on multivariable analysis, age, medical history, duration of CO exposure, duration of coma, degree of poisoning, Interval between ACOP and the first HBOT, total number of HBOTs, and combined rehabilitation treatment were all identified as risk factors for the occurrence of DEACMP.

According to the findings of this study, older patients were at a higher risk of DEACMP—the risk of DEACMP was 8.436 times higher in patients aged ≥ 40 years compared to those aged < 40 years, and the risk of DEACMP was 11.470 times higher in patients with a history of hypertension, diabetes, coronary artery disease, and cerebral infarction than in patients without a medical history. Several studies on DEACMP-related risk factors have also indicated that age and medical history are important variables impacting the occurrence and prognosis of DEACMP^16–18^. These diseases themselves predispose to changes in the blood flow components, resulting in inadequate cerebral blood flow, metabolic problems, and diminished cerebrovascular regulation. Moreover, both cerebrovascular function and central nervous system function gradually decline with age. Therefore, the combined cerebral atherosclerosis, brain metabolic abnormalities, and poor vascular regulation in elderly patients may also lead to aggravation of the disease. Once ACOP occurs, the elderly are at a significantly higher risk of cerebral white matter demyelination than the young, which can be explained by the fact that the repair function of the brain recovers slowly and neuronal damage is continuously exacerbated in the elderly, resulting in a higher risk of DEACMP^18,19^. Consequently, elderly patients with ACOP and with diverse medical histories should be closely followed up while actively undergoing treatment, and the development of DEACMP should be monitored.

The results of our study found that the duration of CO exposure, the duration of coma, and the degree of poisoning were all independent risk factors for the development of DEACMP. Specifically, the risk of DEACMP was 6.328 times higher in patients with 12–24 h of CO exposure than in those with < 6 h of CO exposure, 6.434 times higher in patients with ≥ 24 h of coma than in those with < 6 h of coma, and 16.985 times higher in severe ACOP than in patients with mild ACOP. In other words, longer CO exposure was associated with a greater amount of inhaled CO, longer coma, and more severe poisoning, thereby raising the risk of DEACMP, which may be related to the following mechanism: neuronal necrosis and apoptosis after ACOP. Similarly, prior studies revealed that longer durations of CO exposure and coma were associated with more severe neurological damage, and longer durations of hypoxia were associated with more severe brain damage and demyelination changes in the brain in patients with moderate to severe poisoning, thereby increasing the risk of DEACMP^20–22^. It is clinically more important to actively provide HBOT to patients with more severe conditions or longer durations of CO exposure or coma to improve the hypoxic state of the organism of the patients in time, alleviate the disease, and reduce the risk of DEACMP.

Regarding the ideal period for HBOT treatment, several studies have indicated that HBOT should be administered within 24 h after CO poisoning^21,23,24^. However, another study revealed that the advantage of HBOT after 12 h of CO exposure was not verified, with the greatest benefit occurring within 6 h^25–27^. The benefit of HBOT within 6 h of CO exposure is now routinely acknowledged by a larger number of authors, similar to the findings of our study. In our study, the Interval between ACOP and the first HBOT was < 6 h in 237 cases, accounting for 30.6%. Multivariable analyses revealed that the risk of DEACMP was 3.637 times higher in patients with an interval of ≥ 6 h between the first HBOT and ACOP than in patients with an interval of < 6 h and 7.068 times higher in patients with an interval of ≥ 12 h than in patients with an interval of < 6 h, illustrating that the longer interval was associated with the greater risk of DEACMP. Accordingly, we believe that early HBOT intervention (< 6 h) is beneficial for reducing the incidence of DEACMP, and that patients with ACOP should be treated as soon as feasible if HBOT is allowed, while HBOT should also be actively administered if the interval exceeds 6 h.

Most medical facilities administer HBOT at a pressure of 2–3 ATA. However, there is no precise recommendation for pressured use of HBOT. Birmingham et al.^28^ claimed that inadequate pressure during HBO treatment might only increase oxygen toxicity without providing corresponding benefits. Several randomized controlled studies have demonstrated the effectiveness of HBO treatment with initial treatment pressures exceeding 2.5 ATA. Some reports suggested that treatment pressures below 2.5 ATA might not yield beneficial effects of HBO treatment^29^. However, other studies have shown that HBO treatment at pressures of 2 ATA or 3 ATA can improve overall efficacy^30,31^. There are significant differences among different countries regarding the pressure of hyperbaric oxygen therapy, single oxygen inhalation time, daily treatment frequency, total treatment course, and specific protocols. There is also no completely unified hyperbaric oxygen therapy protocol internationally. Some countries recommend selecting hyperbaric oxygen therapy pressures of 0.24 (or 0.25) to 0.28 MPa for patients who require hyperbaric oxygen therapy, with a single oxygen inhalation time of 60 to 90 min, 1 to 2 treatments within 24 h, and occasionally 3 treatments (with pressure decreasing gradually after the first treatment using higher pressure)^32,33^. Different treatment pressures should be established according to the varying ages and conditions of patients with DEACMP. Univariate analyses in our investigation, revealed that the difference in treatment pressure was not statistically significant.

There is no clear consensus regarding the association between the recommended number of HBOTs and prognosis in patients with ACOP. A retrospective analysis conducted by Huang et al. found that patients who had multiple HBOTs exhibited greater efficacy and had higher survival rates than those who received only one HBOT^15^. In contrast, our data showed that the risk of DEACMP was higher in patients who received > 30 HBOTs was higher than in those who received 1–10 HBOTs. This result may be attributable to the fact that most patients receiving 10 HBOTs were mild to moderate, whereas the majority of patients receiving > 30 HBOTs were severe or even suffered from DEACMP, as well as the presence of multiple confounding factors and large differences in sample sizes between groups. The amount of HBOTs is still debatable, and each hospital has its own HBOT policies for patients with ACOP. The total number of HBOTs depends mostly on the assessment of the condition by clinicians and the personal wishes of patients, so the total number of HBOTs received by ACOP patients is highly arbitrary and varies widely. For different types of patients, the exact number of HBOTs should be decided based on relevant test findings and medical history of patients. The specific treatment protocols for different patients need to be further confirmed by numerous clinical investigations. Identifying DEACMP-related risk factors at an early stage based on the state of patients with ACOP and increasing the number of HBOTs properly can reduce the risk of DEACMP and poor prognostic outcomes of patients^34^.

Patients with ACOP are mostly treated with HBOT clinically, however some patients develop problems and continue to have poor cognitive and motor function recovery and a poor prognosis. Rehabilitative care is a supplement to HBOT and drug therapy. Early and appropriate rehabilitation treatment and training can increase neural excitability, accelerate the establishment of cellular collateral circulation, promote the reorganization or compensation of tissues surrounding the lesion or healthy brain cells, and exert the plasticity of the brain^35^. Multiple studies have demonstrated that the combination of HBOT and comprehensive rehabilitation treatment has a synergistic effect on the treatment of DEACMP and can successfully improve symptoms, survival, and quality of life of the patients^35,36^. In our study, comprehensive rehabilitation treatment was administered to patients who had impaired consciousness, with cognitive and motor dysfunction in the acute phase, to significantly improve cerebral blood circulation, promote consciousness recovery, stimulate neuromuscular cells, increase muscle strength and joint mobility, prevent muscle atrophy, facilitate limb function recovery, and strengthen cognitive function. The rehabilitation treatment involved transcranial direct current stimulation, median nerve electrical stimulation, cerebellum fastigial nucleus electrical stimulation, low frequency pulsed electrical stimulation, and intelligent rehabilitation training for lower limbs. The multivariable analysis revealed that combined rehabilitation treatment as a protective factor against the development of DEACMP. Therefore, we believe that early intervention of rehabilitation treatment for patients who are unconscious, with cognitive and motor dysfunction can be beneficial for restoring the overall functional status of patients and enhancing their poor prognostic outcomes.

## Conclusion

In conclusion, age, medical history, duration of CO exposure, duration of coma, degree of poisoning, HBOT regimen, and combined rehabilitation treatment can aid in early identification of patients with high-risk ACOP who may develop DEACMP, providing a better reference for assessing the condition and risk of patients at admission and enabling the initiation of effective intervention as soon as possible. However, as this is a single-center retrospective study, the analysis of the influencing factors determining the occurrence of DEACMP may not be comprehensive. In addition, the heterogeneity of the patient group complicates the interpretation of the analysis results. Lastly, we did not observe a significant association between CO exposure time ≥ 24 h and the occurrence of DEACMP, possibly due to insufficient sample size in this group. Further multi-center and large-scale prognostic studies are required to provide a more suitable theoretical foundation for directing clinical efforts on prevention and treatment of DEACMP.

## Data Availability

The datasets used and/or analysed during the current study available from the corresponding author on reasonable request.

## Appendix

**Table:**

CO poisoning degree	
Mild	blood carboxyhemoglobin concentration (COHb) may be higher than 10% Any one of the following manifestations: Severe headache, dizziness, limb weakness, nausea, vomiting; Mild to moderate impairment of consciousness without coma
Moderate	blood COHb concentration may be higher than 30% In addition to the above symptoms, the impaired consciousness is manifested as shallow to moderate coma, which is recovered after resuscitation and without obvious complications
Severe	blood COHb concentration may be higher than 50% Any of the following: The degree of impaired consciousness reaches deep coma or decerebral cortex state; Patients with impaired consciousness complicated by any of the following: cerebral edema; shock or severe myocardial damage; pulmonary edema; respiratory failure; upper gastrointestinal hemorrhage; focal cerebral damage such as signs of damage to the pyramidal system or extrapyramidal system
